# Histomorphometric Assessment of Non-Decalcified Plastic-Embedded Specimens for Evaluation of Bone Regeneration Using Bone Substitute Materials—A Systematic Review

**DOI:** 10.3390/ma18010119

**Published:** 2024-12-30

**Authors:** Varvara-Velika Rogova, Stefan Peev, Ralitsa Yotsova, Tsvetalina Gerova-Vatsova, Ivaylo Parushev

**Affiliations:** 1 Department of Oral Surgery, Faculty of Dental Medicine, Medical University of Varna, 9002 Varna, Bulgaria; ralitsa.yotsova@mu-varna.bg; 2 Department of Periodontology and Dental Implantology, Faculty of Dental Medicine, Medical University of Varna, 9002 Varna, Bulgaria; stefan.peev@mu-varna.bg (S.P.); tsvetalina.gerova@mu-varna.bg (T.G.-V.); 3 Department of Clinical Medical Sciences, Faculty of Dental Medicine, Medical University of Varna, 9002 Varna, Bulgaria; ivaylo.parushev@mu-varna.bg

**Keywords:** histology, histomorphometry, undecalcified, non-decalcified, plastic-embedded, bone regeneration, bone substitute, bone graft

## Abstract

With the implementation of bone substitute materials, regeneration strategies have inevitably evolved over the years. Histomorphometry is the optimal means of quantitative evaluation of bone structure and morphology. This systematic review focuses on determining study models, staining methods and histomorphometric parameters used for bone regeneration research on non-decalcified plastic-embedded specimens over the last 10 years. After being subjected to the inclusion and exclusion criteria, 118 studies were included in this review. The results establish the most commonly selected animal model is rat, followed by rabbit, sheep and dog. Strong preference for staining samples with toluidine blue was noted. With regard to histomorphometric parameters, terms related to bone were most frequently assessed, amounting to almost half of recorded parameters. New bone formation was the main descriptor of this category. Residual bone graft and non-bone tissue parameters were also often evaluated. With regard to dynamic histomorphometry, mineral apposition rate (MAR) was the parameter of choice for most researchers, with calcein green being the preferred dye for fluorochrome labelling. An overview of the contemporary literature, as well as weaknesses in the current research protocols have been discussed.

## 1. Introduction

Bone deficiencies present a major challenge for clinicians in many medical fields such as orthopedics, plastic surgery, oral and maxillofacial surgery, otorhinolaryngology and dental implantology. As a result, scientific research has had particular interest in regeneration strategies involving bone substitute materials, with the intention to promote healing and improve patient outcomes [1].

Various materials have been used for bone regeneration, including autogenous bone; bone marrow or plasma derivatives; allogenic cortical and/or cancellous bone blocks or particles; xenogenic products, usually of bovine origin; as well as synthetic grafts such as β-tricalcium phosphate, hydroxyapatite, and many resorbable and non-resorbable polymers [1,2].

The ideal bone substitute displays osteogenic, osteoinductive and osteoconductive properties [3]. Osteogenesis, described as transplantation of osteoblasts capable of bone apposition, is characteristic of autografts. Osteoinduction involves the promotion of bone formation through stimulation of undifferentiated mesenchymal stem cells and their transformation to osteoblasts. It is achieved by bone morphogenic proteins (BMPs) and other growth factors present in allografts. Osteoconduction is direct bone apposition on the surface of a material by the host’s own cells [2]. It is the quality by means of which xenografts and synthetic materials are able to regenerate bone.

Structurally, bone comprises mostly inorganic matter organized as crystal-forming minerals, predominantly hydroxyapatite, as well as collagen type I, forming the organic component. After the deposition of an extracellular matrix by osteoblasts, mineralization of this matrix called osteoid occurs. Human bone is characterized by osteons—concentric lamellar bone around a central Haversian canal that provides blood supply to the cells, with additional Volkmann canals connecting adjacent osteons [4].

An assessment of bone can be performed with several methods. Some non-invasive ones include X-ray-, CT-, MRI- and ultrasound-based modalities [5], as well as vibrational techniques such as Raman spectroscopy [6]. Histological and histomorphometric examinations require an invasive procedure of bone biopsy retrieval.

Histomorphometry allows for quantitative assessment and is regarded as the gold standard for diagnosing metabolic and skeletal bone diseases at the tissue and cellular levels [7]. Specialized computer software is required for measuring quantitative parameters. There are two main types of histomorphometry—static, evaluating bone structure at a particular time point, and dynamic, used for the assessment of bone turnover and resorption rates via florochrome labelling [8]. The latter refers to a technique involving the injection of fluorescent dyes several days prior to assessment, their binding to calcium and subsequent microscopic evaluation of bone formation for the selected period. Often, several fluorochromes are used at different time points. They include tetracycline, alizarin, calcein green and blue, xylenol orange and others [9].

The optimal microscopic method of examination for bone tissue is non-decalcified histology. Decalcification damages tissue and cellular morphology when evaluating hard materials such as implants or ceramics on paraffin-embedded sections, compromising the results [10]. The plastic embedding of bone tissue preserves its microarchitecture and allows for qualitative and quantitative assessment [10].

Several staining methods can be used on non-decalcified specimens. Standard dyes such as hematoxylin and eosin, agents distinguishing non-mineralized osteoid from mineralized bone such as Von Kossa, and Masson–Goldner trichromatic stain are some examples [11]. This ability is also characteristic of toluidine blue, a cationic dye that stains acidic compounds such as mucins, mastocytes, DNA and RNA [4,12]. Due to its metachromatic properties, it has many clinical and laboratory applications.

Different animals have been selected for preclinical bone regeneration research. They include small models such as rodents (rats and mice) and rabbits, as well as large ones like dogs, sheep, goats, pigs and minipigs [13]. The selection of a model is dependent on a number of factors including similarity to human bone in terms of structure and metabolism, defect location with regard to load-bearing, along with practical aspects such as housing and costs [14].

The aim of this systematic review is to describe how regeneration procedures with bone substitute materials are evaluated histomorphometrically on non-decalcified plastic-embedded specimens, identifying study models, staining methods and histomorphometric parameters used for its assessment. To our knowledge, this is the first systematic review that presents the tendencies in histomorphometric protocols for bone regeneration.

## 2. Materials and Methods

### 2.1. Research Question

For the purpose of this systematic review, the following research question was developed, in accordance with the PCC framework:

P (Population)—Animal and human models for studies on bone regeneration with bone substitute materials;

C (Concept)—Histomorphometric parameters;

C (Context)—Articles issued over the last 10 years (2015–2024).

Research question: What histomorphometric parameters (C) are assessed in animal models and human studies on bone regeneration using bone substitute materials (P) in the last 10 years (C)?

### 2.2. Eligibility Criteria

*Inclusion criteria*: research articles in English, published in the last 10 years (2015–2024); articles conducted on non-decalcified plastic-embedded samples after histological staining; articles that report histomorphometric parameters.

*Exclusion criteria*: 1. abstracts, reviews, books, book chapters, case reports and case series; 2. articles without bone substitutes; 3. articles without histomorphometric parameters or only performing semi-quantitative histomorphometric analysis; 4. articles not using plastic embedded specimens; 5. articles that include studies on decalcified specimens only; 6. articles in which histomorphometry is conducted only with SEM; 7. articles discussing intraosseous implants or implant-related parameters; 8. articles from 2014 and earlier; 9. articles in languages other than English.

### 2.3. Information Sources

This systematic review adheres to the Preferred Reporting Items for Systematic Reviews and Meta-Analyses (PRISMA) Statement guidelines [15]. It was registered in the INPLASY database (DOI:10.37766/inplasy2024.8.0086) and can be found at https://inplasy.com/inplasy-2024-8-0086/, accessed on 18 August 2024.

A comprehensive electronic search for research articles in the Scopus, Web of Science and PubMed databases was conducted on 6 August 2024.

### 2.4. Search Strategy

Only full-sized research articles in English were included. The search strategy comprised an advanced search in the selected databases. The keywords that were used for the respective databases are as follows:

1. Scopus: ((histology) OR (histomorphometry) OR (histomorphometric)) AND ((undecalcified) OR (non-decalcified)) AND ((bone AND substitute) OR (bone AND graft) OR (bone AND replacement AND material)) AND PUBYEAR > 2014 AND PUBYEAR < 2025 AND (LIMIT-TO (DOCTYPE, “ar”)) AND (LIMIT-TO (LANGUAGE, “English”)).
2. Web of Science: ALL = (((histology) OR (histomorphometry) OR (histomorphometric)) AND ((undecalcified) OR (non-decalcified)) AND ((bone substitute) OR (bone graft) OR (bone replacement material))).
3. PubMed: (((histology) OR (histomorphometry) OR (histomorphometric)) AND ((undecalcified) OR (non-decalcified)) AND ((bone substitute) OR (bone graft) OR (bone replacement material))).

Expanded search: ((“anatomy and histology”[MeSH Subheading] OR (“anatomy”[All Fields] AND “histology”[All Fields]) OR “anatomy and histology”[All Fields] OR “histology”[All Fields] OR “histology”[MeSH Terms] OR “histologies”[All Fields] OR “histomorphometry”[All Fields] OR (“histomorphometric”[All Fields] OR “histomorphometrical”[All Fields] OR “histomorphometrically”[All Fields] OR “histomorphometrics”[All Fields])) AND (“undecalcified”[All Fields] OR “non-decalcified”[All Fields]) AND (“bone substitutes”[MeSH Terms] OR (“bone”[All Fields] AND “substitutes”[All Fields]) OR “bone substitutes”[All Fields] OR (“bone”[All Fields] AND “substitute”[All Fields]) OR “bone substitute”[All Fields] OR (“bone transplantation”[MeSH Terms] OR (“bone”[All Fields] AND “transplantation”[All Fields]) OR “bone transplantation”[All Fields] OR (“bone”[All Fields] AND “graft”[All Fields]) OR “bone graft”[All Fields]) OR (“bone substitutes”[MeSH Terms] OR (“bone”[All Fields] AND “substitutes”[All Fields]) OR “bone substitutes”[All Fields] OR (“bone”[All Fields] AND “replacement”[All Fields] AND “material”[All Fields]) OR “bone replacement material”[All Fields]))) AND (y_10[Filter]).

### 2.5. Study Selection and Data Collection

Titles and abstracts were screened for eligibility by two independent reviewers (V.R. and R.Y.). Publication data (titles, abstract, authors and year of publication) were exported to an Excel file. Duplicates were removed, and the full-text studies were subjected to the eligibility criteria. Tables were created, containing the following information: authors and publication year, animal model (for in vivo studies), bone substitute material (and trade name when applicable), barrier membrane (if used), implantation period, staining method and histomorphometric parameters. Disagreements between the reviewers were resolved through discussion and consultation with a third reviewer (S.P.) when a uniform decision could not be made. Inter-rater levels of agreement were quantitatively measured with Cohen’s kappa. A value of 0.766 was calculated, indicating substantial agreement between the researchers [16].

### 2.6. Risk of Bias Assessment

For the purpose of this review, a quality assessment was conducted using the SYRCLE ROB tool for animal studies and Cochrane RoB2 [17] tool for human studies. The results were visualized with the ROBVIS tool [18]. Risk of bias was independently evaluated by two reviewers, V.R. and R.Y.

## 3. Results

The initial search identified 578 studies with potential relevance to the topic from the three respective electronic databases. After the exclusion of 81 duplicate records, 497 articles remained. Then, the articles that did not meet the eligibility criteria were excluded. 

Finally, after thorough inspection, 118 studies were included in the current review.

**Figure 1:**
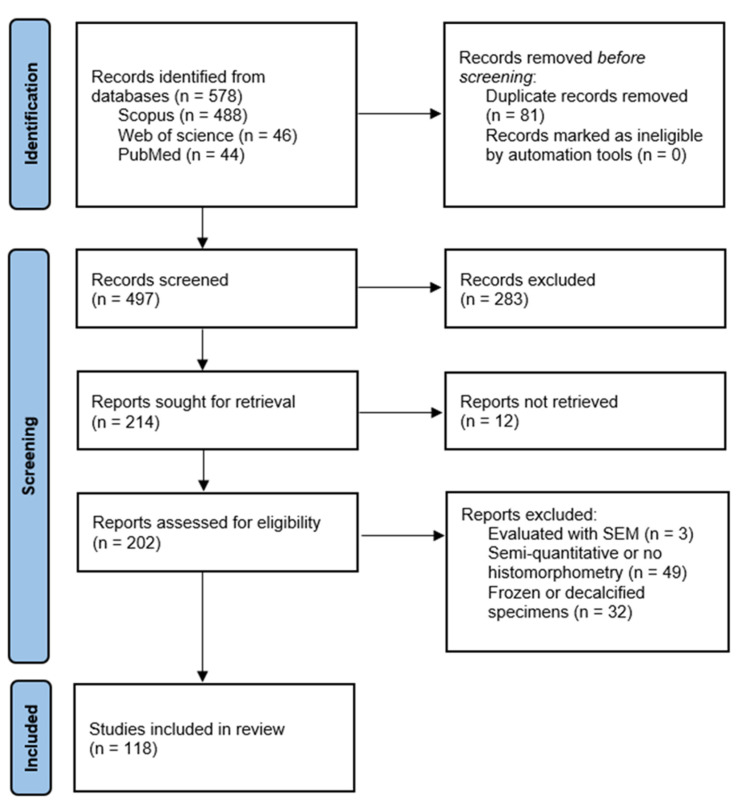
PRISMA diagram of the research.

Figure 1 represents a PRISMA flow chart of the study selection process [15].

### 3.1. Animal Studies

In our review, 98 animal studies were included (Table 1). Various biomaterials and bone substitutes have been used to assist bone regeneration. Table 1 summarizes the characteristics of each study, including references; authors; animal model; sample size; bone substitute material and trade name, if applicable; use of a barrier membrane; implantation period; staining; and histomorphometric parameters evaluated. MS Office Excel 2019 was used for data synthesis and presentation.

**Figure 2:**
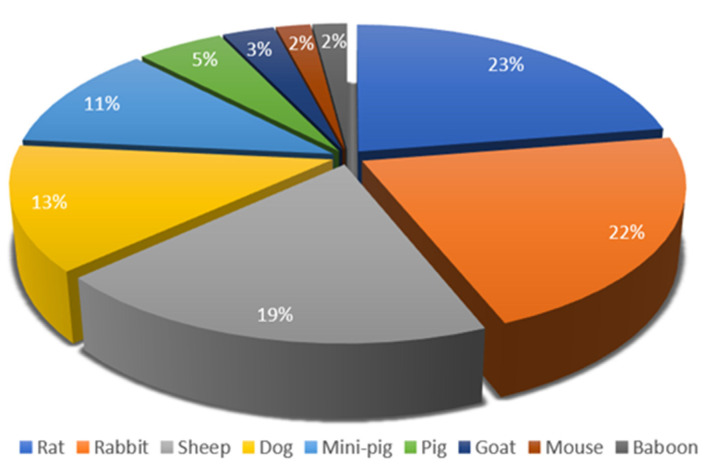
Animal models.

Different animal models have been used for studying bone regeneration. In our review, the most commonly used model was rat (n = 22), closely followed by rabbit (n = 21) and sheep (n = 19). Fewer experiments have been conducted on dogs (n = 13), minipigs (n = 11) and pigs (n = 5). Other models include goats (n = 3), mice (n = 2) and baboons (n = 2). Figure 2 depicts the distribution of each model in this review.

### 3.2. Human Studies

In this review, 20 eligible human trials were included, as illustrated in Table 2.

**Figure 3:**
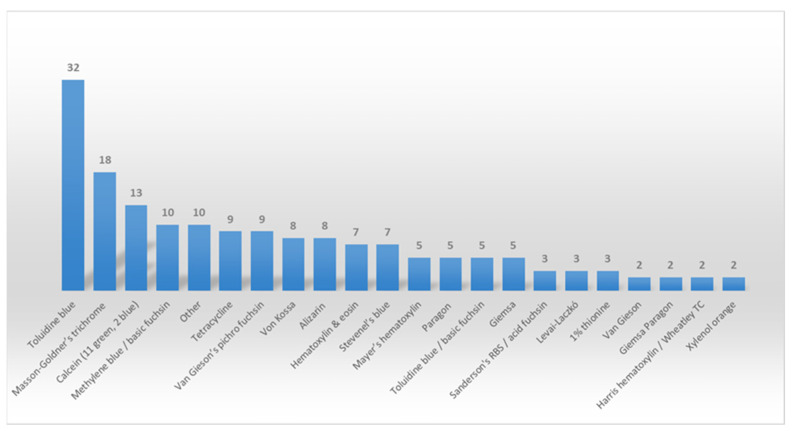
Staining methods ranked by the number of studies that utilize them.

Several staining methods have been used for bone evaluation (Figure 3). It is evident that toluidine blue is the preferred dye among most researchers. The “other” category includes dyes mentioned once—Jenö and Géza staining, Safranin-Orange, mixture of one-part MacNeal’s tetrachrome (methylene blue, azure II and methyl violet) and two-part toluidine blue, trichrome stain according to Plenck, 1% toluidine blue dissolved in 1% borax and mixed with 1% pyronin G, Alcian blue, Villanueva–Goldner, Movat penta-chrome, Pyronine Y, Azure II and pararosaniline.

### 3.3. Histomorphometric Parameters

**Figure 4:**
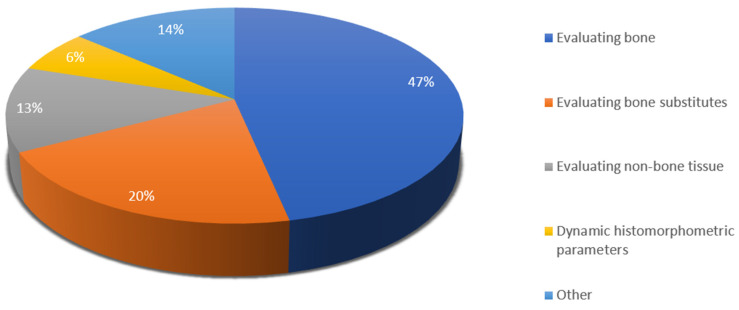
Histomorphometric parameters recorded in the reviewed literature.

Among the 118 studies included in this review, 349 histomorphometric parameters have been evaluated. Of those, 162 parameters (47%) characterize new bone tissue, 71 (21%) are related to residual bone substitute particles, 44 (13%) discuss non-bone tissue and 50 (13%) are concerned with different, infrequently mentioned parameters. A total of 22 of the parameters were dynamic histomorphometry, which amounts to 6%. The forementioned distribution is illustrated in Figure 4.

**Figure 5:**
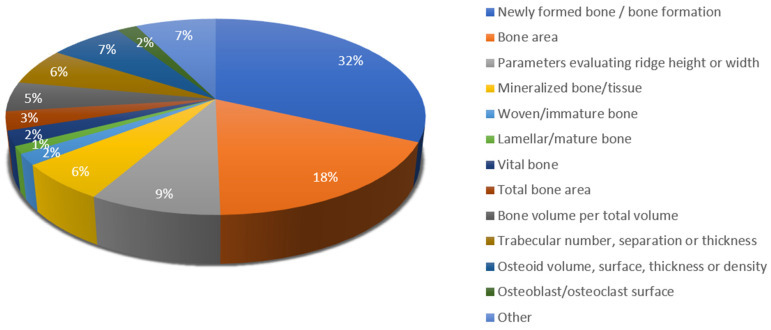
Terms for histomorphometric parameters evaluating bone.

Figure 5 further subdivides the parameters related to bone formation and their distribution. Among them, the most commonly used parameter is new bone formation, which has been given different terms depending on the study—newly formed bone, (new) bone formation, bone area, etc.

**Figure 6:**
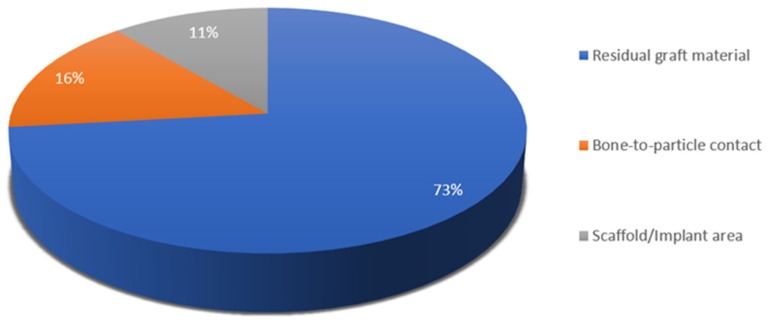
Terms for histomorphometric parameters evaluating bone substitute materials.

The parameters characterizing bone substitute materials (Figure 6) consist of three groups—residual material particles, bone-to-particle (bone-to-biomaterial) contact and scaffold (or sometimes labelled “implant”) area.

**Figure 7:**
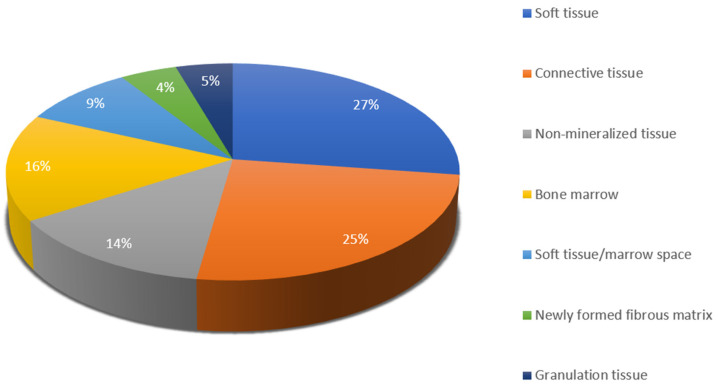
Terms for histomorphometric parameters evaluating non-bone tissue.

Figure 7 illustrates terms that represent non-bone tissue.

**Figure 8:**
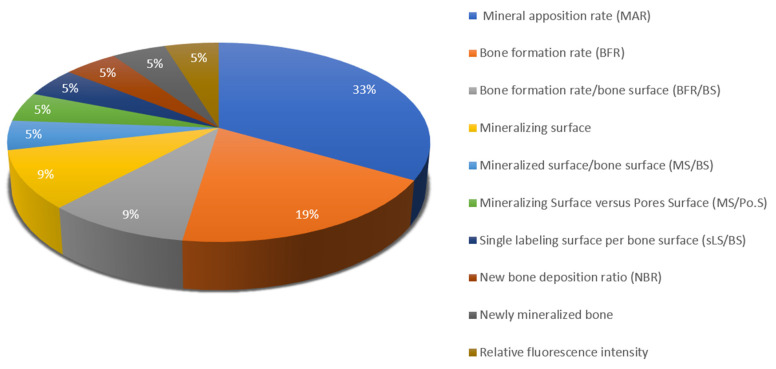
Dynamic histomorphometric parameters.

In addition to static measurements, some of the studies also include dynamic histomorphometry measurements. Figure 8 shows all dynamic histomorphometric parameters mentioned in the reviewed literature, as well as their distribution.

Other, less frequently occurring histomorphometric parameters not related to the forementioned can be divided into several categories. The biggest proportion consists of the parameters related to defect dimensions—defect diameter, remaining defect width, remaining/initial defect width, defect closure and final distance scaffold–defect margin. This category is followed by terms jointly describing newly formed bone, residual bone substitute and soft tissue areas or percentage of augmented area; bone regeneration area; total grafted area; total ROI; neo-borne tissue volume; tissue area; and total area of the grafted area. The combined parameters of bone and bone substitute material are mineralized tissue, gained tissue and binary area. Another group of parameters is those measuring blood vessel number, diameter, volume or percentage. Several other parameters mentioned once or twice can be seen in the tables.

Several studies that appeared to meet the eligibility criteria were excluded after assessment. These include decalcified specimens [137,138,139,140,141,142,143,144,145,146,147,148,149,150,151,152,153,154], as well as non-decalcified but frozen or cryosections [155,156,157,158,159,160,161,162].

### 3.4. Risk of Bias Assessment

**Figure 9:**
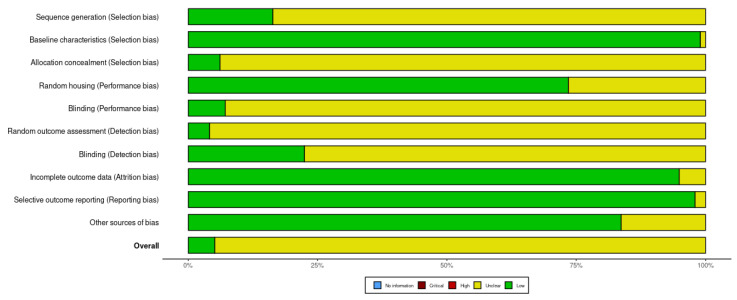
Risk of bias assessment for animal studies.

For animal studies (Figure 9), the results reveal low risk of selection bias for baseline characteristics, as well as low attrition and reporting bias. For other domains, however, most of the studies do not provide sufficient information regarding sequence generation, allocation concealment, blinding and random outcome assessment, as these categories have been marked as “unclear”. Overall, it is unlikely that these domains affect histomorphometric assessment, as it is calculated with computed software based on objective microscopic measurements.

**Figure 10:**
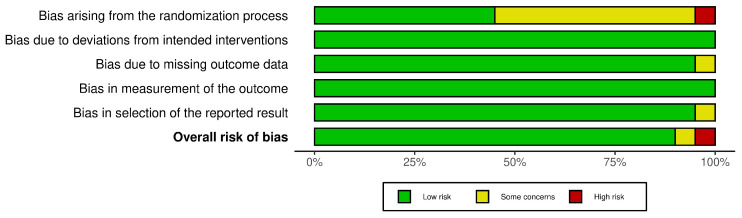
Risk of bias assessment for human studies.

With regard to human studies (Figure 10), the overall risk of bias was assessed as low. “Some concerns” arising in several studies were due to insufficient data regarding the randomization process (lack of computer-generated randomization, no information about concealment, etc.). Only one of the twenty studies presented a high risk of bias due to dubious randomization protocol.

Traffic light charts for risk of bias assessment of individual studies can be found in the Supplementary Materials, as well as the PRISMA checklist.

## 4. Discussion

Bone regeneration procedures are performed in many medical fields, from joint replacement orthopedics, maxillofacial reconstructions, plastic surgery and otorhinolaryngology to novel specialties like dental implantology. With the aim of optimizing healing, various bone substitute materials have been created.

Assessing the effectiveness of novel materials or medical devices requires vigorous preclinical testing. In vitro studies are the basis of scientific experiments but are not sufficient when evaluating biological processes in living organisms. Several in vivo models have been used for bone regeneration research, with each having its advantages and drawbacks [13].

The findings of this systematic review suggest that small animal models such as rat and rabbit are preferred among researchers. Both species are relatively easy to handle and require lower costs compared to larger animals, hence their selection. When it comes to disadvantages, rodents and rabbits have significant differences in bone structure and load-bearing mechanics when compared to humans [163,164]. A major limitation of rat bones is their size [165], as well as the lack of a Haversian canal system found in humans [13]. Rabbit bone is also dissimilar to human bone in terms of microstructure and vascular network but shares a resemblance in terms of its bone mineral density [166]. Rabbit bone turnover is also faster than human bone turnover, as these animals reach skeletal maturity at around 6 months of age [164,166].

Large animal models such as sheep, dogs and pigs have more similarity to humans in terms of bone structure, metabolism and load-bearing but are more expensive and harder to house and care for [164,167]. They are usually the last preclinical model prior to human testing [168].

Preferred locations for creating bone defects in both small and large animal models are long bones such as tibia and femur [165]. Rat calvaria, ovine scapula, dog mandibles and pig cranial bones are also utilized [13]. Selection of the implantation site should ideally complement the clinical and biomechanical conditions being researched [14], but as evidence suggests, this is not always possible.

As can be seen in the results, various implantation periods have been implemented, ranging from a few days to several months. These depend on the animal model, its age, its bone turnover rate, as well as the stage of healing that is being analyzed [14].

It is believed that barrier membranes are crucial for the success of guided bone regeneration procedures, as they prevent the ingrowth of rapidly proliferating epithelium and connective tissue and allow for bone in-growth in the defect area [169]. Nonetheless, guided bone regeneration without a barrier membrane has been attempted. Ma et al. [69] compared guided bone regeneration with a barrier membrane and with periosteum for the treatment of alveolar bone defects in dogs. The authors found superior bone formation with periosteum coverage and highlighted its osteogenic potential and key role in bone remodeling. This notion, however, has been disputed by other authors [170]. Deng et al. [171] conducted a study with periosteum as a barrier membrane in 40 patients and reported “acceptable” results in terms of bone regeneration. A limitation of their study, however, is the omission of a control group or comparison with guided bone regeneration with barrier membranes.

Our findings demonstrate that in 30 out of the 98 animal studies (30,61%), the bone defect filled with bone substitute material is covered by a barrier membrane. It should be noted that most animal studies are conducted on long bones (tibia and femur) or calvaria, not on alveolar bones, and defects are not in contact with the oral cavity. Among the 20 human studies, 11 included a barrier membrane and 5 collagen products in the form of a sponge, sheet or matrix covered the defect. This supports the notion that some sort of isolation between the oral cavity and bone substitute material is desired [169].

Animal testing serves as a “bridge” between preclinical and clinical trials, a prominent example of translational research. On the subject of this review, studies on animal models investigate the mechanisms of bone regeneration with bone substitute materials, assessing the effects of the latter on living tissues. As can be seen from the results, various experimental materials have been tested in vivo and only a few of them have been translated “from bench to bedside”. This exemplifies the vigorous and extensive testing of material safety, efficacy and properties required before warranting approval for clinical research.

When it comes to staining undecalcified bone, a wide variety of methods and dyes can be used. Our findings ascertain toluidine blue as the most commonly utilized stain, either alone or combined with other dyes such as basic fuchsin or pyronin yellow. One of its main advantages is metachromasia—the ability to stain different tissue components with varying color intensity, different from that of dyes [12]. With regard to bone tissue, it allows for distinction between mineralized bone matrix, osteoid, and soft tissue [172]. Moreover, its ability to stain cement lines helps researchers distinguish between newly formed and old bone, thereby evaluating and quantifying bone formation [10]. Other commonly used dyes that can differentiate mineralized from non-mineralized tissue are Goldner’s trichromatic stain, as well as methylene blue/basic fuchsin and Stevenel’s blue/Van Gieson’s picro fuchsin combinations [10,147,173]. Masson–Goldner’s trichromatic stain, the second most commonly used staining method, includes the application of three dyes, with the third being used as a counterstain [174]. Its major drawback is the complexity of its protocol, as it includes 3 dyes and 13 steps to perform [174], whereas toluidine blue and its modifications require 6 to 9 steps and consist of only one dye [10,175]. The solutions applied for Goldner’s trichrome also have multiple components, increasing the intricacy of protocol [176]. Its binding to and respective staining of collagen fibers may also be variable, potentially leading to inconsistencies [174]. Additionally, for the purposes of non-decalcified plastic-embedded bone histology, modifications of Masson–Goldner’s trichrome may be needed to improve distinction between mineralized components [177,178,179]. Modifications according to the embedding media have also been discussed, depending on the chemical composition of the resin—methyl-methacrylate, glycol-methacrylate or epoxy resin [179].

Bone histomorphometry allows for a quantitative assessment of bone formation, remodeling and metabolism on the cellular, as well as tissue levels [8].

The region of interest (ROI) is an important component in histomorphometric evaluation. Although not clearly defined in the literature, it has been described as “the area of the slide to be studied” [180]. The ROI can drastically vary depending on the type of intervention being studied; for example, it could be one or several areas surrounding an entire dental implant [181,182] or just a region at certain threads [183]. In bone regeneration studies, the region of interest is not always thoroughly described. It could be presumed that the entire defect area is the region of interest in most of these cases, as often, histomorphometric parameters are presented as fractions or percentages of the whole defect [52,78,81]. Some animal studies establish a particular region of interest by selecting it with a geometrical shape with well-defined dimensions—a rectangle [37,71,80,87,94,110,125], square [65,126] or circle [20,32,39,59,62,95,96,109,113]. The vast majority of human studies employ a cylindrical region of interest. This is because most core biopsies on humans are taken with a trephine drill, and its diameter and length are what determine the dimensions of the ROI.

Various histomorphometric parameters have been used in the reviewed literature. They can be divided into three main categories—parameters related to bone, non-bone tissue and residual graft material. For the sake of completeness, we included two more groups, as we wanted to account for all parameters discussed in the studies. These are dynamic histomorphometric parameters and “other”, which combines terms unrelated to the abovementioned, as well as several combined parameters.

With regard to bone, it can be seen that the majority of studies discuss at least one bone-related parameter. Different terms have been proposed, but the most established are newly formed bone or bone formation, highlighting the word “new”. Other parameters such as bone area, mineralized tissue and bone volume per total volume do not discuss whether the bone is newly formed or old, which could cause confusion and lead to misunderstanding. Schulz et al. [135] and Hemmerlein et al. [50] differentiate woven and lamellar bone, which have structural and functional differences. Woven bone or immature bone is disorganized, with randomly ordered collagen fibers and lower mineralization, while lamellar or mature bone is characterized by collagen fibers arranged in lamellae [184]. In four studies [122,123,129,136], the term vital bone is proposed, without clear explanation of its meaning; presumably, it pertains to bone containing cells such as osteocytes or osteoblasts. Measurements have been calculated in micrometers or millimeters, or as a fraction (percentage) of the whole region of interest. Few studies strictly follow the proposed terminology established by the American Society of Bone and Mineral Research (ASBMR) [185], likely due to its complexity. Examples of such parameters are bone volume per total volume (BV/TV), trabecular thickness (Tb.Th), trabecular separation (Tb.Sp), trabecular number (Tb.N), osteoid volume per bone volume (OV/BV), osteoid surface per bone surface (OS/BS), osteoid thickness (O.Th), eroded surface per bone surface (ES/BS), etc.

When it comes to the description of bone substitute materials, fewer terms have been used. The objective of applying most bone grafts is space maintenance, which will be filled with bone once the material resorbs; hence, the ideal bone substitute has resorption rates that match the recipient’s bone formation rates [186]. Some biomaterials such as calcium phosphate resorb quickly, whereas others like hydroxyapatite are considered practically non-resorbable [187]. This is why a parameter like residual graft material or particles is valuable in histomorphometric assessment. Moreover, material resorption can be evaluated in studies analyzing bone substitute materials at several time points, as the values of this parameter decrease. Scaffold area is a similar term, with a particular reference to the shape of the graft. Bone-to-particle or bone–biomaterial contact denotes the connection between the material and newly formed bone, or in other words, new bone apposition on the graft. Several authors measure this parameter as “percentage of the length of bone contact to graft surface to the circumference of the graft particle” [27,38,53]. Bone–biomaterial contact analyzes the osteoconductive properties of the materials, where they act as scaffolds for osteoblasts to depose new bone on their surface [27,38]. Osteoconduction is a characteristic quality of xenogenic and alloplastic (synthetic) materials, which are the most commonly utilized grafts.

Bone grafting is inevitably accompanied by connective tissue proliferation, either physiologically occurring in the healing process or as a foreign body reaction [188]. Due to the wide variety of terms and lack of clear definitions, we derived the term “non-bone tissue” and included all corresponding parameters in this category. Synonyms were also grouped together in the subdivisions (e.g., marrow space and medullary space, connective tissue and stromal tissue, etc.). This was necessary because terms were phrased differently but had similar meanings. As can be seen from the results, soft tissue, connective tissue, bone marrow and non-mineralized tissue are the most frequently used. Bone marrow and marrow spaces are found in the trabecular spaces of spongy bone, hence the establishment of these parameters. The term “non-mineralized” tissue has been used by several authors [24,26,28,42,117] but is unclear what it refers to—osteoid, connective tissue or other. Embryonically, blood vessels are also made of connective tissue, but due to their rare occurrence in studies and clear distinction, rather than connective tissue, we classified them in “other parameters”.

Dynamic histomorphometric parameters are not as prevalent as static ones, owing to their complexity of execution as they require fluorochrome injection several days and/or application at several timepoints prior to assessment, as well as additional costs for equipment and qualification. Moreover, the main application of dynamic histomorphometry is in diagnosing metabolic bone diseases, rather than bone regeneration studies [189]. Nonetheless, calcein green, tetracycline and alizarin are fluorochromes used for evaluating mineralization fronts in the animal studies included in this review. Corresponding parameters include mineral apposition rate (MAR), bone formation rate (BFR) and mineralizing surface (MS). Dynamic histomorphometric parameters measure bone turnover over a particular time period [190]. One or more dyes may be applied, creating single, double or multiple labelled surfaces [191]. Mineral apposition rate is measured as the mean distance between two double labels divided by the time between their application [192]. Bone formation rate equals the quantity of bone deposited on a particular bone surface per year [192]. Mineralizing surface measures the bone perimeter exhibited by a single or a double labelled assay, evaluating mineralization fronts [192,193].

This work presents some valuable clinical implications and their potential influence on research and practice. Comprehension of histomorphometric results facilitates clinicians’ critical thinking and analysis of the properties of different bone substitute materials and helps them select the optimal material for each individual case. Properties like resorption rate and bone-forming ability are of utter importance and their understanding would help answer questions: How quickly and to what extent does a particular material resorb? Is the vacated space filled with bone or soft tissue? How much time does bone need to form and remodel after regeneration with bone substitutes and what is the quality of this bone? What is the influence of each bone substitute material on bone healing? How long after bone regeneration can implants be placed?

As a future clinical prospect, nanoparticles (NPs) can also be added to biomaterials to potentially improve material qualities, as well as the biological response to the material. Intrinsically therapeutic NPs (ITNPs) are molecules that are able to trigger bioactive activities upon interaction with biological entities [194]. They reportedly have antioxidant, anti-inflammatory, antiangiogenic and antibacterial effects and are also able to act as drug delivery agents. The prospect of discovering molecules with other properties, for example, those that promote bone regeneration, is immense [194].

When discussing the limitations of this review article, it should be mentioned that a meta-analysis was not conducted. The rationale for this decision was partly rooted in the review question and partly due to the heterogeneity of research protocols among studies. Our aim was to describe, analyze and summarize current strategies in bone regeneration research. Comparisons between different animal models and especially healing times, various materials, as well as parameters that do not always correspond to each other, are likely to diminish the integrity of a meta-analysis and lead to drawing conclusions with questionable credibility, owing to differences in methodology.

## 5. Conclusions

This systematic review is an overview of the existing literature from the last decade regarding bone regeneration with bone substitute materials, performed on resin-embedded specimens. It established the most commonly used animal model (rat), staining method (toluidine blue) and histomorphometric parameter (new bone formation).

Formulation of clear definitions for the regions of interest and histomorphometric parameters, adherence to the established nomenclature, as well as unification of terminology could improve the current state of research on bone regeneration.

## Figures and Tables

**Table 1:** Characteristics of animal studies.

Ref.	Authors	Model	Sample Size	Bone Substitute Material (Trade Name— If Applicable)	Barrier Membrane	Implantation Period	Staining	Histomorphometric Parameters
[19]	Ahmed et al. (2020)	Rat	40	BCP (Maxresorb^®^) Hyaluronic acid gel (Hyadent^®^) BCP (Maxresorb^®^) combined with hyaluronic acid gel (Hyadent^®^)	No	4 and 10 weeks	Methylene blue and basic fuchsin	Newly formed bone area (NF-BA, %); Remaining BCP particles (R-BCP, %)
[20]	Akay et al. (2020)	Rat	80	Oxytocin-loaded: - β-TCP/HA hydrogel - PLGA sustained-release microspheres - PLGA microspheres + hydrogel graft	No	4 and 8 weeks	Masson Goldner’s trichrome	New bone formation (%); Residual graft (%)
[21]	Al-Asfour et al. (2017)	Rabbit	8	Demineralized human dentin onlay graft Autogenous bone block	No	12 weeks	Toluidine blue	Newly formed bone (%); Connective tissue (%)
[22]	Alayan et al. (2016)	Sheep	9	Collagen-stabilized bovine bone (Bio-Oss^®^ Collagen) DBBM (Bio-Oss^®^) combined with autogenous bone	Yes	8 and 16 weeks	Toluidine blue	Newly formed bone (NB, %); Residual graft particles (RG, %); Soft tissue and marrow spaces (STM, %); Particle osseointegration (OI, %)
[23]	Alt et al. (2016)	Goat	3	Synthetic HA injectable paste (Ostim^®^) HA (Ostim^®^) combined with porcine collagen	No	6 weeks	Hematoxylin and eosin	New bone formation (%)
[24]	Aludden et al. (2020)	Mini-pig	24	DBBM (Bio-Oss^®^) DBBM (Bio-Oss^®^) combined with autogenous bone at different ratios Autogenous bone block	Yes	10, 20 and 30 weeks	Toluidine blue	Percentage of bone (POB, %); Non-mineralized tissue (NMT, %); Graft material (DBBM, %)
[25]	Alvira-González et al. (2016)	Dog	18	β-TCP (KeraOs^®^) β-TCP (KeraOs^®^) with fibronectin β-TCP (KeraOs^®^) with fibronectin and autologous adipose-derived stem cells (ADSCs)	Yes	1, 2 and 3 months	Jenö and Géza staining method	Bone regeneration area (mm^2^); Neo-formed bone matrix (%); Collapsed surface (%); Medullary space (%); Biomaterial contact to the neo-formed matrix (%)
[26]	Baker et al. (2015)	Mouse	36	Nanocomposites consisting of organically modified montmorillonite clay dispersed in PDLA, with or without added rhBMP-2	No	2, 4 and 6 weeks	Goldner’s trichrome	Mineralized tissue (%); Non-mineralized tissue (%)
[27]	Barbieri et al. (2017)	Dog	8	CaP/alkylene oxide copolymer putty material (AttraX Putty^®^) CaP/BioGlass™/ collagen composite material (Vitoss™)	No	12 weeks	Methylene blue and basic fuchsin	Bone formation (%); Bone-to-particle contact (%); Resorption rate (%)
[28]	Benic et al. (2016)	Dog	8	DBBM (Bio-Oss^®^) DBBM block (Bio-Oss^®^ Spongiosa Block) Collagen-containing cancellous equine-derived bone block	Yes	4 months	Toluidine blue	Augmented area (AA) within the former bone defect (mm^2^); Mineralized tissue (MT), non-mineralized tissue (NMT), and residual bone substitute (BS) within AA (mm^2^); Bone substitute to mineralized tissue contact surface (BS-MT surface, %); Horizontal thickness of the augmented area and mucosa (HT, mm)
[29]	Brockmeyer et al. (2015)	Mini-pig	12	PEG hydrogel blocks	No	4 and 12 weeks	Smith– Karagianes staining (methylene blue/alizarin red S)	Volume density (VD) of newly formed bone (%)
[30]	Brockmeyer et al. (2015)	Mini-pig	12	β-TCP (Calciresorb^®^) alone or with added rhGDF-5	Yes	4 and 12 weeks	Toluidine blue	Volume density (VD) of newly formed bone (%)
[31]	Bungartz et al. (2017)	Sheep	44	CPC reinforced with PLGA fibers and containing rhGDF-5	No	3 and 9 months	Masson Goldner’s trichrome Oxytetracycline and alizarin red	Bone volume/total volume (BV/TV); Trabecular thickness (Tb.Th); Trabecular number (Tb.N); Osteoid volume/bone volume (OV/BV); Osteoid surface/bone surface (OS/BS); Osteoid thickness (O.Th); Eroded surface/bone surface (ES/BS); Mineralized surface/bone surface (MS/BS); Mineral apposition rate (MAR); Bone formation rate/bone surface (BFR/BS)
[32]	Camargo et al. (2023)	Rat	42	DBBM (Bio-Oss^®^) with per os administration of alendronate	No	4 and 12 weeks	Methylene blue and basic fuchsin	New bone formation (%)
[33]	Catros et al. (2015)	Mini-pig	14	PEG scaffold with or without osteogenic protein-1 (OP-1)	No	3 weeks	Basic fuchsin/ toluidine blue	Bone surface (%); Bone height (mm); Osteoid density (mm/mm^2^)
[34]	Chang et al. (2017)	Rat	16	Gelatin-HA/β-TCP cryogel composite Cryogel composite infused with BMP-2 Cryogel composite infused with PLGA microspheres encapsulating BMP-2	No	28 days	Calcein green and alizarin complex-non	New bone deposition ratio (NBR, %)
[35]	Chen et al. (2021)	Rat	32	3D-printed PCL scaffold with or without plasma-rich-fibrin	No	4 and 8 weeks	Hematoxylin and eosin	Tissue area (mm^2^); Bone area (%); Connective tissue area (%); Residual PCL area (%)
[36]	Dau et al. (2016)	Mini-pig	18	DBBM (BioOss^®^) Synthetic HA (NanoBone^®^) Synthetic HA (Ostim^®^)	No	5 weeks and 8 months	Toluidine blue	Residual bone substitute material (%); Soft tissue (%); Newly formed bone (%)
[37]	de Barros et al. (2017)	Dog	8	DBBM (MinerOss^®^ X)	No	12 weeks	Alizarin red	Buccal bone crest level (BCL, mm); Alveolar ridge width (ARW, mm); Connective tissue (CT, %); Total bone area (TBA, %); Residual graft particles (RGP, %)
[38]	de Carvalho et al. (2019)	Mini-pig	5	Three experimental DBBM bone substitutes	Yes	3 months	Methylene blue and basic fuchsin	Regeneration % (proportion of ROI2/ROI1); Newly formed bone (%), biomaterial (%) and soft tissue (%) within the overall defect area (ROI1); Bone-to-material contact (%) in the augmented area (ROI2)
[39]	Diogo et al. (2024)	Rabbit	12	Marine collagen and apatite scaffolds (from blue shark skin and teeth) Bovine collagen/ synthetic apatite scaffolds	No	12 weeks	Levai–Laczkó	Newly formed bone-like tissue (%); Trabecular separation (mm); Trabecular thickness (mm); Bone mineral density (g/cm^3^)
[40]	Delgado-Ruiz et al. (2018)	Dog	6	BCP (Straumann^®^ Bone Ceramic)	Yes	2 months	Levai–Laczko	Bony contour (BC, mm^2^); Penetration of the graft material into the socket (PPG, %); New bone (NB, %); Residual graft (RG, %); Connective tissue (CT, %)
[41]	Elschner et al. (2017)	Rat	84	Collagen-stabilized bovine bone (Bio-Oss^®^ Collagen) alone or with added undifferentiated or osteogenically differentiated mesenchymal stem cells (MSCs)	No	6, 9 and 12 weeks	Masson–Goldner’s trichrome	Newly formed bone (BV, %); Remaining defect width (rDW, %)
[42]	Escoda-Francolí et al. (2018)	Rat	30	β-TCP (KeraOs^®^) β-TCP (KeraOs^®^) combined with fibronectin	Yes	6 and 8 weeks	Levai–Laczkó	Defect diameter, mm; Target area, mm^2^; Area of mineralized bone matrix (MBM, mm^2^ and %); Non-mineralized tissue (NMT, mm^2^ and %); Residual bone substitute (BS, mm^2^ and %); Augmented area (MBM+NMT+BS) within the former bone defect (AA, mm^2^ and %); Gained tissue (MBM+BS, %)
[43]	Evrard et al. (2024)	Mini-pig	3	Native and decellularized porcine bone blocks	No	3 months	Hematoxylin and eosin, Alcian blue, and Von Kossa Calcein green and tetracycline	Number of osteoclasts; Fluorochrome positive surface area per total surface area (%)
[44]	Fischer et al. (2015)	Dog	2	Collagenated porcine bone graft (Gen-Os^®^) with added pamidronate (Aredia^®^)	No	4 months	Van Gieson’s pichro fuchsin	Horizontal alveolar bone width (mm); Buccal bone level (mm); Lingual bone level (mm)
[45]	Foley et al. (2021)	Sheep	41	Autogenous bone flap fixed with bone adhesive composed of tetra-calcium phosphate and phosphoserine (TTCP-PS)	No	12 weeks, 1 and 2 years	Modified paragon	Implant area (%); Bone area (%); Soft tissue area (%)
[46]	Friedmann et al. (2022)	Dog	5	DBBM (BioOss^®^) Calcium sulfate (3D Bond™)	Yes	3 months	Toluidine blue or paragon	Total ROI (newly formed tissue within the augmented area, %); Residual non-bone graft area within the ROI (%); ∆ ROI—non-bone graft area (%); Horizontal ridge thickness at different levels (mm)
[47]	Grassmann et al. (2015)	Rabbit	24	Autogenous cancellous bone graft	No	3 and 6 weeks	Toluidine blue	New bone formation (μm^2^ and %)
[48]	Hedenqvist et al. (2020)	Rabbit	20	3D-printed CPC implant Autogenous bone	Yes	12 weeks	Toluidine blue	Total area, cm^3^; Binary area (graft material and bone, cm^3^); Fraction of binary area to total area (%)
[49]	Hernández-Suarez et al. (2021)	Rabbit	5	Silicon combined with autogenous bone and PRF membrane Silicon with only PRF or autogenous bone	No	3 weeks	Von Kossa 5% silver nitrate and calcein	Bone height (BH, mm—only on VK); Bone area (BA, µm^2^ and %); Bone perimeter (BP, µm)
[50]	Hemmerlein et al. (2024)	Rabbit	42	3D-printed scaffolds made of Calcium magnesium phosphate cements Magnesium phosphate cements	No	6, 12, 24 and 30 weeks	Toluidine blue	Scaffold material (%); Woven bone (%); Lamellar bone (%); Cartilage tissue (%)
[51]	Hung et al. (2020)	Mini-pig	6	BCP (Bicera^®^) DBBM (Bio-Oss^®^)	No	12 weeks	Masson’s trichrome Alizarin complexnon and calcein green	Bone area per total tissue area (BA/TA, %); Bony surface per bone area (BS/BA, %); Residual particles (%); Newly formed bone (%); Connective tissue (%); Single labeling surface per bone surface (sLS/BS, %); Mineralization apposition rate (MAR, 10^−2^ μm/day)
[52]	Hutchens et al. (2016)	Sheep	unclear	SiCaP SiCaP combined with an autogenous iliac crest bone graft SiCaP mixed with bone marrow aspirate	No	4, 8 and 12 weeks	Modified paragon	Absolute bone volume (BV/TV, %); Absolute graft volume (GV/TV, %); Normalized bone volume [BV/(TV-GV), %]
[53]	Intapibool et al. (2021)	Pig	8	Autogenous bone BCPs with different HA/β-TCP ratios: - commercially available BCP (MBCP+™) - BCP with HA30 - BCP with HA70	No	4, 8, 12 and 16 weeks	Toluidine blue and basic fuchsin	New bone formation (%); Residual material particles (%); Bone-to-graft contact (%)
[54]	Kaiser et al. (2022)	Sheep	14	Magnesium cements: Struvite (MgNH_4_PO_4_.6H_2_O) K-struvite (MgKPO_4_.6H_2_O)	No	2 and 4 months	Paragon Tetracycline and calcein green	Relative amounts of residual cement (Cm.Ar/T.Ar, %); Newly formed bone (B.Ar/T.Ar, %); Soft tissue per total tissue area (ST.Ar/T.Ar, %); Bone formation rate (BFR, µm^3^/µm^2^/d)
[55]	Kaiser et al. (2023)	Sheep	14	Magnesium cements: Oxychloride (Mg_3_(OH)_5_Cl∙4H_2_O) Amorphous Mg phosphate Newberyite (MgHPO_4_∙3H_2_O)	No	2 and 4 months	Giemsa Tetracycline and calcein green	Relative amounts of residual cement (Cm.Ar/T.Ar, %); Newly formed bone (B.Ar/T.Ar, %); Soft tissue per total tissue area (ST.Ar/T.Ar, %); Bone formation rate (BFR, µm^3^/µm^2^/d)
[56]	Kanjilal et al. (2021)	Rat	unclear	Human DBM DBM + ZnCl2 DBM + Zn stearate Human bone chips Zn-bound human bone chips	No	12 weeks	Stevenel’s blue and Van Gieson’s picro fuchsin	New bone area (mm^2^)
[57]	Kauffmann et al. (2021)	Mini-pig	18	PDLLA/CaCO_3_ composite loaded with rhBMP2 and rhVEGF165	No	4 and 13 weeks	Toluidine blue	New bone formation (mm^2^); Bone density (%)
[58]	Kluge et al. (2019)	Sheep	13	Autogenous bone “dust” Bioactive glass (Bonalive^®^)	No	3 weeks	Alizarin complexion	Newly mineralized bone (%)
[59]	Knabe et al. (2019)	Sheep	36	Si-CAOP (Osseolive^®^) Si-TCP (Ceracell^®^) TCP (Cerasorb^®^ M)	No	2 weeks, 1, 3, 6, 12 and 18 months	Mayer’s hematoxylin	Bone area fraction (%); Particle area fraction (%); Bone–particle contact (%)
[60]	Knabe et al. (2023)	Rat	80 *	Si-CAOP scaffolds infiltrated with stem cells	Yes	3 and 6 months	Mayer’s hematoxylin	Bone area fraction (%); Scaffold material area fraction (%); Bone–bioceramic contact (%)
[61]	Korn et al. (2017)	Rat	84	Collagen-stabilized bovine bone (BioOss^®^ Collagen) alone or with added mesenchymal stem cells (MSCs)	No	6, 9 and 12 weeks	Masson–Goldner trichrome	Remaining defect width (%); New bone formation (%)
[62]	Kowalewicz et al. (2022)	Rabbit	36	3D-printed calcium magnesium phosphate cement scaffolds with alkaline or acidic post-treatment TCP scaffolds	No	6, 12 and 24 weeks	Toluidine blue	Scaffold material (%); Bone (%); Soft tissue (%)
[63]	Kumagai et al. (2019)	Rabbit	10 *	β-TCP blocks (Affinos^®^)	No	2 and 6 weeks	Villanueva–Goldner	Length and average diameter of the largest vessels (μm); Length of newly formed bone (μm)
[64]	Kunert-Keil et al. (2015)	Rat	24 *	BCP (calc-I-oss™ CRYSTAL) β-TCP (calc-i-oss™ CLASSIC)	No	4 weeks	Masson–Goldner trichrome	Regenerated bone (%)
[65]	Ledet et al. (2018)	Goat	14	PEEK interbody cage filled with autogenous bone	No	14 and 18 weeks	Stevenel’s blue and Van Gieson’s picro fuchsin	Bone area fraction (%)
[66]	Lee et al. (2015)	Rat	72	β-TCP/hyaluronic acid hydrogel alone or loaded with rhBMP-2	No	5 and 9 weeks	Hematoxylin and eosin	Newly formed bone length (%)
[67]	Li et al. (2016)	Sheep	16	Baghdadite scaffolds (calcium–zirconium– silicate) Baghdadite scaffolds modified with a PCL coating containing bioactive glass nanoparticles	No	26 weeks	Toluidine blue	Total defect length crossed by bridging bone (%); Total area of bridging bone (mm^2^); Maximum distance of bone infiltration (mm^2^); Total area of infiltrating bone (mm^2^)
[68]	Liu et al. (2018)	Rabbit	18	Mussel-inspired CPC with and without added polydopamine (PDA)	No	2, 4, and 8 weeks	Calcein, tetracycline and xylenol orange	Relative fluorescence intensity (IOD units)
[69]	Ma et al. (2016)	Rabbit	30	β-TCP scaffolds	No	1, 2, 4, 8, and 12 weeks	Van Gieson’s picro fuchsin	Blood vessel volume (BVV, %); Neo-borne tissue volume (NTV, %); Residual material volume (RMV, %); Number and diameter (μm) of vessels; Proportion of tissue penetration (PTP, %)
[70]	Maenz et al. (2017)	Sheep	40	CPC CPC reinforced with PLGA	No	3 and 9 months	Trichrome stain according to Plenck	Bone volume/total volume (BV/TV, %), Trabecular thickness (Tb.Th, μm); Trabecular number (Tb.N, 1/mm); Osteoid volume (OV/BV, %); Osteoid surface (OS/BS, %); Osteoid thickness (O.Th, μm); Eroded surface (ES/BS, %)
[71]	Maia et al. (2015)	Dog	8	Collagen-stabilized bovine bone (BioOss^®^ Collagen)	No	12 weeks	Alizarin red Alizarin red, tetracycline, calcein green and blue	Buccal crest level (BCL, mm); Alveolar ridge width (ARW, mm); Connective tissue (CT, %); Total bone area (TBA, %); New bone area (NBA, %); Residual graft particles (RGP, %); Mineralization rate (%)
[72]	Major et al. (2022)	Sheep	6	3D-printed aluminum oxide ceramic (LithaLox 350D) Ti-6Al-4V porous scaffold	No	12 weeks	1% thionine	New bone formation (%)
[73]	Martinez et al. (2015)	Rabbit	12	DBBM (Bio-Oss^®^) β-TCP (KeraOs^®^)	Yes	1, 4, 8, 16, 32 and 52 weeks	Harris hematoxylin and Wheatley’s trichrome	Bone (%); Graft material (%); Soft tissue (%)
[74]	Mehl et al. (2016)	Mini-pig	4	DBBM (Endobon^®^) DBBM (Endobon^®^) with autogenous bone	Yes	6 months	Toluidine blue	Bone (% and mm^2^); Bone marrow (% and mm^2^); Residual graft material (% and mm^2^); Soft tissue (% and mm^2^)
[75]	Moest et al. (2020)	Pig	18	DBBM (BioOss^®^)	Yes	4, 8 and 12 weeks	Toluidine blue O	Newly formed bone (NFB, %); Residual graft (DBBM, %); Soft tissue (ST, %)
[76]	Morra et al. (2015)	Rabbit	10	BCP with or without porcine collagen nanolayer coating of the particles	No	12 weeks	Oxytetracycline	Mineral apposition rate (MAR, μm/day); Bone formation rate (BFR/B.Pm, μm^2^/μm/day)
[77]	Moschouris et al. (2017)	Rat	14	Collagen sponge doped with recombinant Wnt3a protein	Yes	4 and 7 days	Toluidine blue	Defect closure (%); Mineralized tissue surface per defect length (mm^2^/mm); Non-mineralized tissue surface per defect length (mm^2^/mm)
[78]	Moya et al. (2016)	Dog	5	Resorbable ceramic rods based on antimicrobial soda-lime glass powder (SiO_2_–Na_2_O–Al_2_O_3_–CaO–B_2_O_3_)	No	4 months	Harris hematoxylin and Wheatley trichrome	New bone formation (%)
[79]	Ortiz-Puigpelat et al. (2019)	Dog	9	Three BCP block grafts with different HA/β-TCP ratios (HA100, HA75 and HA50)	Yes	4, 12 and 24 weeks	Toluidine blue and fuchsin	Area of newly formed bone (NB, %); Area of residual graft material (RM, %); Area of connective tissue (CT, %)
[80]	Park et al. (2015)	Dog	24 *	BCP (OSTEON™ II) soaked in rhBMP-2	No	4 and 12 weeks	Toluidine blue and basic fuchsin	New bone volume (%); New cementum length (%); New connective tissue attachment (mm); Osteoclast count
[81]	Pripatnanont et al. (2016)	Rabbit	16	BCP with different HA/β-TCP ratios (9:1 and 8:2) Autogenous bone chips	No	2 and 8 weeks	Masson–Goldner’s trichrome	New bone area (%); Particle material area (%)
[82]	Ramazanoglu et al. (2021)	Pig	20	BCP (Straumann^®^ Bone Ceramic) in PEG hydrogel with or without added porcine mesenchymal stem cells (MSCs) Autogenous bone	No	1, 2, and 4 weeks and 3 months	Toluidine blue O	Newly formed bone (%); Graft material (%)
[83]	Richter et al. (2023)	Rat	46	Strontium-modified CPC scaffolds Sr-CPC + mesoporous bioactive glass (MBG) Sr-CPC + MBG + hypoxia conditioned medium	No	6 and 12 weeks	Masson–Goldner trichrome	Remaining/initial defect width (%); Bone formation (%); Final distance scaffold–defect margin (μm)
[84]	Ripamonti et al. (2015)	Baboon	6	Coral-derived CaCO_3_ scaffolds alone or loaded with hTGF-β3 and/or hNoggin protein	No	2 and 3 months	Methylene blue basic fuchsin and toluidine blue	Newly formed bone (%)
[85]	Ripamonti et al. (2016)	Baboon	10	Biphasic CaCO_3_/HA scaffolds, with or without hTGF-β3	Yes	3 months	Methylene blue/basic fuchsin and modified Goldner’s trichrome	Bone formation (%)
[86]	Roman et al. (2015)	Dog	5	Collagen matrix (Mucograft^®^), covered with a collagen membrane	Yes	5 months	Paragon	Total buccal bone height (BBH, mm); Total lingual bone height (LBH, mm); LBH–BBH (mm); Buccal and lingual bone widths (mm), measured at 1, 3 and 5mm from the crest; Soft tissue area (mm^2^)
[87]	Russmueller et al. (2015)	Rabbit	16	3D-printed scaffolds: Acrylates Vinylesters Vinylcarbonates	No	12 weeks	1% thionine	Newly formed bone (%); Bone-to-implant contact (BIC, %)
[88]	Russmueller et al. (2015)	Sheep	18	PLA scaffolds (PolyMax^®^) filled with β-TCP (ChronOS^®^) with added: Coagulation factor XIII (Fibrogammin-P^®^) Autologous bone marrow Venous blood	No	12 weeks	1% thionine	Newly formed bone (%); Residual biomaterial (%); Marrow space (%)
[89]	Sachse et al. (2023)	Sheep	48	HA/β-TCP/DCPD cylinders (Conduit^TM^) coated with rhBMP-2 or rhGDF-5	No	3, 6 and 9 months	Oxytetracycline	Mineralizing surface (%)
[90]	Schmidt et al. (2022)	Rabbit	40	Magnesium scaffolds with: MgF_2_ coating MgF_2_+CaP coating	No	6, 12, 24 and 36 weeks	Toluidine blue O	Areas of scaffold, bone, gas, granulation tissue, osteoid, plate, bone marrow and cartilage (%)
[91]	Schneppendahl et al. (2016)	Rabbit	48	Autogenous bone graft combined with PRP and/or hyperbaric oxygenation therapy	No	3 and 6 weeks	Toluidine blue	New bone formation (μm^2^ and %)
[92]	Schorn et al. (2021)	Pig	14	BCP (BEGO OSS S^®^) BCP injectable paste (BEGO OSS S inject^®^) BCP embedded in porcine collagen (Flexbone, Botiss) Autogenous bone particles	Yes	4 and 8 weeks	Toluidine blue	New bone formation (NBF, %); Newly formed fibrous matrix (FM, %); Residual bone substitute material (BSM, %)
[93]	Schorn et al. (2021)	Pig	14	DBBM: Sintered (BEGO OSS^®^) and non-sintered (BioOss^®^) Autogenous bone	Yes	4 and 8 weeks	Toluidine blue	New bone formation (NBF, %); Newly formed fibrous matrix (FM, %); Residual bone substitute material (BSM, %)
[94]	Schröter et al. (2023)	Sheep	14	Magnesium phosphate cement paste containing: Struvite (MgNH_4_PO_4_·6H_2_O)Dittmarite (MgNH_4_PO_4_·H_2_O) Farringtonite (Mg_3_(PO_4_)_2_) Newberyite (MgHPO_4_·3H_2_O)	No	2 and 4 months	Giemsa Tetracycline and calcein green	Relative amount of residual cement (Cm.Ar/T.Ar, %); Newly formed bone (B.Ar/T.Ar, %); Soft tissue per total tissue area (ST.Ar/T.Ar, %); Bone formation rate (BFR, µm^3^/µm^2^/d)
[95]	Shaheen et al. (2021)	Rat	24	DBBM (InterOss^®^) with pre- or post-surgical i.v. administration of zolendronic acid (ZA)	No	6 weeks	Methylene blue and basic fuchsin	Bone area (%); Remaining bone graft (%)
[96]	Shaheen et al. (2021)	Rat	80 *	β-TCP (Kasios^®^ TCP) with s.c. coadministration of alendronate and simvastatin	No	3 weeks	Methylene blue and basic fuchsin	Bone area (BA, %); Remaining bone graft (RBG, %)
[97]	Sheftel et al. (2020)	Sheep	10	Equine collagen cone (PARASORB Cone^®^) Equine collagen cone reinforced with BCP particles (PARASORB Cone Oss^®^) Bovine bone matrix coated with PLA/PCL copolymer DBBM (Bio-Oss^®^)	Yes	16 weeks	Mixture of one-part MacNeal’s tetrachrome (methylene blue, azure II and methyl violet) and two-part toluidine blue	Residual graft (RG, %); Newly formed bone (NB, %); Soft tissue and marrow spaces (CT, %)
[98]	Shen et al. (2021)	Goat	24	PEEK cage filled with autogenous bone	No	24 weeks	Stevenel’s blue and Van Gieson’s picro fuchsin	Trabecular bone area fraction (%)
[99]	Shi et al. (2020)	Rat	18	Scaffolds made of PLGA and β-TCP, with or without incorporated icaritin (ICT)	No	4 and 8 weeks	Goldner’s Trichrome Xylenol orange and calcein green	Osteoblast surface per bone surface (Ob.S/BS); Osteoid surface (Os.Pm, mm); Osteoid surface per bone surface (Os.S/BS, %); Osteoid area (Os.Ar, mm^2^); Mineralizing surface (MS, mm); Mineral apposition rate (MAR); Bone formation rate per bone surface (BRF/BS)
[100]	Staedt et al. (2020)	Rabbit	40	DBBM (Bio-Oss^®^) DBBM (Bio-Oss^®^) covered with a collagen membrane	Yes	1, 3, 7, 14 and 21 days	Toluidine blue	Newly formed bone (%)
[101]	Steiner et al. (2021)	Mini-pig	4 *	DBBM (Creos™ xenogain)	Yes	12 weeks	Sanderson’s RBS and counter-stained with acid fuchsin	Bone area (%); Non-bone tissue area (connective tissue and empty space, %); Granules area (%)
[102]	Stigler et al. (2019)	Sheep	5	β-TCP (CeraSorbM^®^) β-TCP (CeraSorbM^®^) modified with stromal cells and/or nano-diamond particles (ND) β-TCP (CeraSorbM^®^) with ND and autogenous bone	Yes	3 and 6 months	Toluidine blue	Bone formation (%)
[103]	Su et al. (2015)	Rabbit	20	β-TCP scaffolds alone or with added human osteoprotegerin (hOPG)-transfected periodontal ligament stem cells (PDLSCs)	Yes	12 weeks	Toluidine blue	Bone area (%)
[104]	Sudheesh Kumar et al. (2018)	Rabbit	4	Biphasic PCL scaffold with incorporated hyaluronic acid hydrogel loaded with rhBMP-2, placed in a PLLA dome	Yes	8 weeks	Hematoxylin and eosin	New bone fill (%)
[105]	Susin et al. (2018)	Rat	140	Bovine HA: BioOss^®^ SmartBone^®^ Cerabone^®^ 403Z013 403Z014 Synthetic HA/β-TCP: Reprobone^®^ Fortoss Vital^®^ Ceraball^®^ SynOss^®^ Cerament^®^ Human allografts: cortical, cancellous and cortico-cancellous bone (Creos^®^)	Yes	4 weeks	Sanderson’s RBS and counter-stained with acid fuchsin	Linear defect closure (%); Bone area fraction (%); Biomaterial area fraction (%);
[106]	Susin et al. (2022)	Rat	80	Bovine HA: Bio-Oss^®^ Cerabone^®^ DirectOss^®^ Ceraball^®^ 403Z013 403Z014 Synthetic HA/ß-TCP: Reprobone^®^	Yes	8 weeks	Sanderson’s RBS and counter-stained with acid fuchsin	Linear defect closure (%); Bone area fraction (%); Biomaterial area fraction (%)
[107]	Thieu et al. (2023)	Dog	8	DBBM (Bio-Oss^®^) TiO_2_-scaffold (Corticalis AS)	Yes	4 and 12 weeks	Movat penta-chrome and Von Kossa/Van Gieson	Collagen (%); Extracellular matrix mineralization (%)
[108]	Torbjörn et al. (2021)	Rabbit	20	Calcium phosphate hollow implant Autogenous bone particles	Yes	12 weeks	1% toluidine blue dissolved in 1% borax and mixed with 1% pyronin G	Bone area (mm^2^); Graft material area (mm^2^); Fraction filled with new bone/graft material (%)
[109]	Van Houdt et al. (2018)	Rat	12	Calcium phosphate cement (α-TCP) and PLGA (Purasorb^®^) alone or loaded with alendronate (ALN)	No	4 and 12 weeks	Methylene blue and basic fuchsin	Bone area (%); Material remnants (%);
[110]	Van Houdt et al. (2021)	Rat	56	Hydrogel of bisphosphonated hyaluronan/CaP nanoparticles alone and with high or low dosages of BMP-2 (InductOs^®^)	No	12 weeks	Methylene blue and basic fuchsin	Bone area (%)
[111]	Voss et al. (2022)	Sheep	24	Titanium scaffold Biodegradable PDLLA/CaCO_3_ scaffold Polyethylene scaffolds with two pore sizes PMMA-based bone cement block	No	3, 6 and 12 months	Giemsa (transversal plane) and Safranin-Oange/ Von Kossa (sagittal plane)	Mineralized bone (MdB/TAr and MdB/PZ, %); Connective tissue (CoT/TAr and CoT/PZ, %); Scaffold area (Sc/TAr and Sc/PZ, %); Void area (Vd/TAr and Vd/PZ, %)
[112]	Witek et al. (2020)	Sheep	6	PLGA scaffolds with or without leukocyte- and platelet-rich fibrin (L-PRF)	No	6 weeks	Stevenel’s blue and Van Gieson’s picro fuschin	Bone area fraction occupancy (BAFO, %)
[113]	Wüster et al. (2024)	Sheep	36	Synthetic HA (Osbone^®^) Bovine-derived HA (Bio-Oss^®^) β-TCP (Cerasorb^®^ M)	No	2 weeks, 1, 3, 6, 12 and 18 months	Mayer’s hematoxylin, Giemsa and Von Kossa stains, modified according to Gross and Strunz	Newly formed bone (%); Residual graft material (%); Bone–biomaterial particle contact (%)
[114]	Zakaria et al. (2016)	Rabbit	16	Synthetic HA (GranuMas^®^) HA granules (GranuMas^®^) combined with PRP Autograft	No	6 and 16 weeks	Masson–Goldner trichrome	New bone area (%)
[115]	Zamuner et al. (2021)	Mouse	9	Wollastonite-Diopside ceramic scaffolds with peptide biofunctionalized surface	No	45 days	Goldner’s Trichrome and Von Kossa Calcein green	Mineralized surface versus pore surface (Md.S/Po.S, %); Mineralizing surface versus pore surface (MS/Po.S, %); Mineral apposition rate for single label (MAR-SL, μm/day); Number of vessels (<2, 2–4, >4)
[116]	Zhang et al. (2022)	Rat	20	Calcium phosphate silicate (Ca_5_(PO_4_)_2_SiO_4_) doped with iron	No	12 weeks	Calcein green and alizarin red	Mineralization rate (MAR, µm/d)

NOTE: This table includes static and dynamic histomorphometry, with dynamic underlined; * not all samples are undecalcified. HA = hydroxyapatite; β-TCP = β-tricalcium phosphate; BCP = biphasic calcium phosphate; CaP = calcium phosphate; CPC = calcium phosphate cement; CaCO_3_ = calcium carbonate; DBBM = deproteinized bovine bone mineral; DBM = demineralized bone matrix; Si-CAOP = silicon containing calcium alkali orthophosphate; SiCaP = silicate-substituted calcium phosphate; DCPD = dicalcium phosphate dihydrate/brushite; PLA = polylactic acid/polylactide; PLLA = poly-l-lactic acid; PLGA = polylactic-co-glycolic acid/poly(D, L-lactide-co-glycolide); PDLLA = poly(D,L-lactic acid); BMP = bone morphogenic protein; rhBMP = recombinant human bone morphogenic protein; rhGDF = recombinant human growth differentiation factor; rhVEGF = recombinant human vascular endothelial growth factor; hTGF = human transforming growth factor; PCL = poly(ε-caprolactone); PEEK = polyether ether ketone; PMMA = polymethyl methacrylate; PEG = polyethylene glycol; PRF = platelet-rich fibrin; PRP = platelet-rich plasma.

**Table 2:** Characteristics of human studies.

Ref.	Authors	Sample Size	Bone Substitute Material (Trade Name— If Applicable)	Barrier Membrane	Implantation Period	Staining	Histomorphometric Parameters
[117]	Aludden et al. (2024)	18	DBBM (Bio-Oss^®^) DBBM (Bio-Oss^®^) combined with autogenous bone chips	Yes	10 months	Toluidine blue	Total area of the grafted area (%); Total bone area (%); Total area of DBBM (%); Total area of non-mineralized tissue (NMT, %)
[118]	Areewong et al. (2019)	33	PRF plug Blood clot	No	2 months	Toluidine blue and basic fuchsin	New bone formation (%)
[119]	Belouka et al. (2016)	44	Nanocrystalline HA (Ostim^®^) Nanoporous HA (NanoBone^®^)	Yes	6 months	Giemsa	Bone (%); Bone substitute (%); Soft tissue (%)
[120]	Berberi, Nader (2016)	11	Mineralized cortical bone allograft (Puros^®^ Cortical)	Yes	4 months	Giemsa Paragon	Bone marrow (%); Mineralized bone (%); Woven bone (%); Residual particles (%)
[121]	Canullo et al. (2015)	20	Mg-enriched HA granules (SINTlife^®^) mixed with blood and covered with a collagen sponge (Gingistat^®^)	No	4 and 12 months	Toluidine blue and Pyronine Y	Bone (%); Residual biomaterial (%); Medullary spaces (%); Blood vessels (%)
[122]	Crespi et al. (2016)	7	Collagen sheets (Condress^®^)	No	4 months	Toluidine blue	Vital bone (%); Connective tissue (%)
[123]	Crespi et al. (2016)	15	Collagen sheets (Condress^®^)	No	3 months	Toluidine blue	Vital bone (%); Connective tissue (%)
[124]	Göttsche et al. (2021)	13	Autologous bone flap	No	12–457 months	Masson–Goldner, Kossa/Van Gieson and toluidine blue	BV/TV (%)
[125]	Knabe et al. (2017)	120	β-TCP (CEROS^®^ TCP)	Yes	6 months	Mayer’s hematoxylin	Bone area fraction (%)
[126]	Knabe et al. (2023)	38	Si-CAOP (Osseolive^®^) β-TCP (CEROS^®^)	Yes	6 months	Mayer’s hematoxylin	Bone area fraction (%); Graft material particle area fraction (%); Bone–biomaterial contact (%)
[127]	Kohal et al. (2015)	51	DBBM (BioOss^®^) soaked in blood	Yes	3 and 6 months	Tolonium chloride (toluidine blue)	Mineralized bone in the augmented area (%); Bone substitute material in the augmented area (%); Mineralized bone in the pristine bone (%)
[128]	Maiorana et al. (2017)	7	DBBM (Bio-Oss^®^) covered with a collagen matrix (Mucograft^®^)	No	6 months	Azure II and pararosaniline	Newly formed bone (%); Residual graft material (%); Mineralized bone (%); Connective tissue and bone marrow (%)
[129]	Nishimoto et al. (2019)	21	DBBM (Bio-Oss^®^) Equine bone mineral (Equimatrix^®^) Mineralized cancellous human bone particles (OSSIF-Isem™)	Yes	8–12 months	Stevenel’s blue and Van Gieson picro fuchsin	Vital bone (VB, %); Residual bone materials (RBM, %); Connective tissue/ marrow (CT, %)
[130]	Pierre et al. (2022)	20	Mixture of 50:50 cortical and cancellous mineralized human bone (Puros^®^ Cortical Cancellous Particulate Allograft)	Yes	6 months	Giemsa–Paragon	Newly formed bone (%); Residual bone graft (%)
[131]	Pripatnanont et al. (2017)	7	Autogenous bone blocks combined with DBBM particles (Bio-Oss^®^), covered with PRF and a collagen barrier membrane	Yes	4–6 months	Hematoxylin and eosin	Total bone area (%)
[132]	Pripatnanont et al. (2022)	16	BCP FDBA	No	12 weeks	Hematoxylin and eosin and Masson–Goldner’s trichrome	New bone area (%); Residual graft area (%)
[133]	Rolvien et al. (2020)	46	Human cancellous bone chips	No	-	Toluidine blue, von Kossa and trichrome Masson–Goldner	Bone volume per tissue volume (BV/TV, %); Trabecular number (Tb.N, 1/mm); Trabecular thickness (Tb.Th, µm); Trabecular separation (Tb.Sp, µm); Osteoblast surface per bone surface (Ob.S/BS, %); Osteoclast surface per bone surface (Oc.S/BS, %); Number of osteocytes per bone area (N.Ot/B.Ar, 1/mm^2^); Number of osteocyte canaliculi per osteocyte lacuna (N.Canaliculi/ Ot.Lc, #)
[134]	Saito et al. (2021)	45	β-TCP/PLGA moldable material (Easy-graft^®^) FDBA (LifeNet Health^®^) covered with a collagen dressing (CollaPlug^®^)	No	20 weeks	Stevenel’s blue and Van Gieson’s picro fuchsin	Mineralized tissue (MT, %); Remaining bone grafting material (RBM, %); Non-mineralized tissue (NMT, %)
[135]	Schulz et al. (2016)	17	Collagen-stabilized bovine bone (Bio-Oss^®^ Collagen) Autogenous bone block	Yes	6 months	Masson– Goldner trichrome	Mature bone (%); Immature bone (%); Total bone (%); Stromal tissue (%); Remaining bone substitute (%)
[136]	Sivolella et al. (2020)	16	DBBM (Endobon^®^) DBBM (Bio-Oss^®^)	Yes	4 months	Stevenel’s blue and Van Gieson’s picro fuchsin	New bone formation (%); Vital bone (%); Marrow or fibrous tissue (%); Residual bone graft (%)

HA = hydroxyapatite; β-TCP = β-tricalcium phosphate; BCP = biphasic calcium phosphate; DBBM = deproteinized bovine bone mineral; FDBA = freeze-dried bone allograft; Si-CAOP = silicon containing calcium alkali orthophosphate; PLGA = polylactic-co-glycolic acid/poly(D, L-lactide-co-glycolide); PRF = platelet-rich fibrin.

## Data Availability

The original contributions presented in this study are included in the article/Supplementary Materials. Further inquiries can be directed to the corresponding author.

## Supplementary Materials

The following supporting information can be downloaded at: https://www.mdpi.com/article/10.3390/ma18010119/s1, Figure S1: Traffic light chart for risk of bias assessment for animal studies; Figure S2: Traffic light chart for risk of bias assessment for human studies; Table S1: Prisma Checklist.
