# Supplementary figures and images for: Histomorphometric Assessment of Non-Decalcified Plastic-Embedded Specimens for Evaluation of Bone Regeneration Using Bone Substitute Materials—A Systematic Review
Source: Materials (Basel). 2024 Dec 30;18(1):119. doi: 10.3390/ma18010119 (PMC11722015; doi:10.3390/ma18010119)

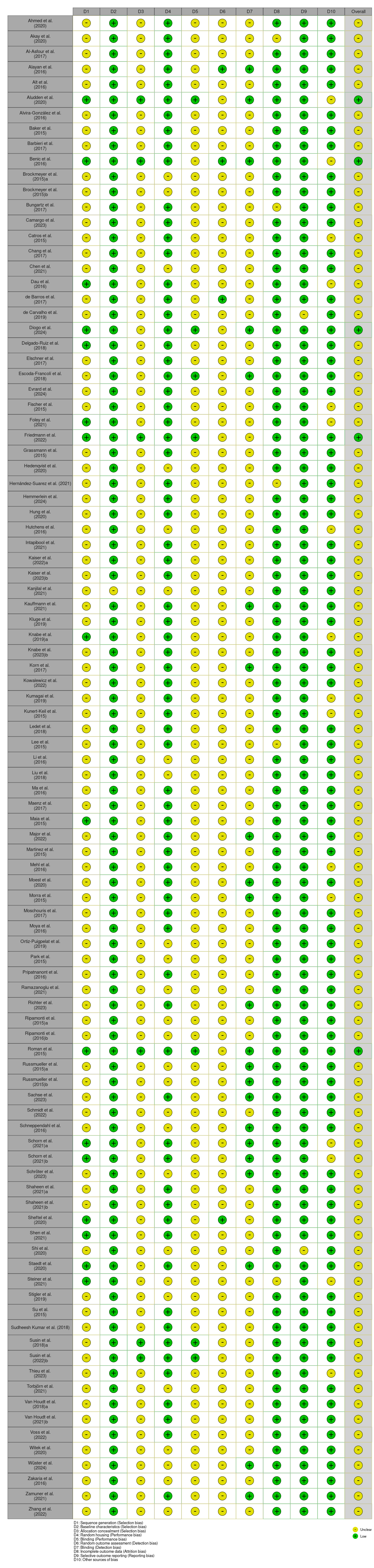

Supplement: Supplementary file 1 [file materials-18-00119-s001.zip › Supplementary file 1 - risk of bias assessment for animal studies.png]

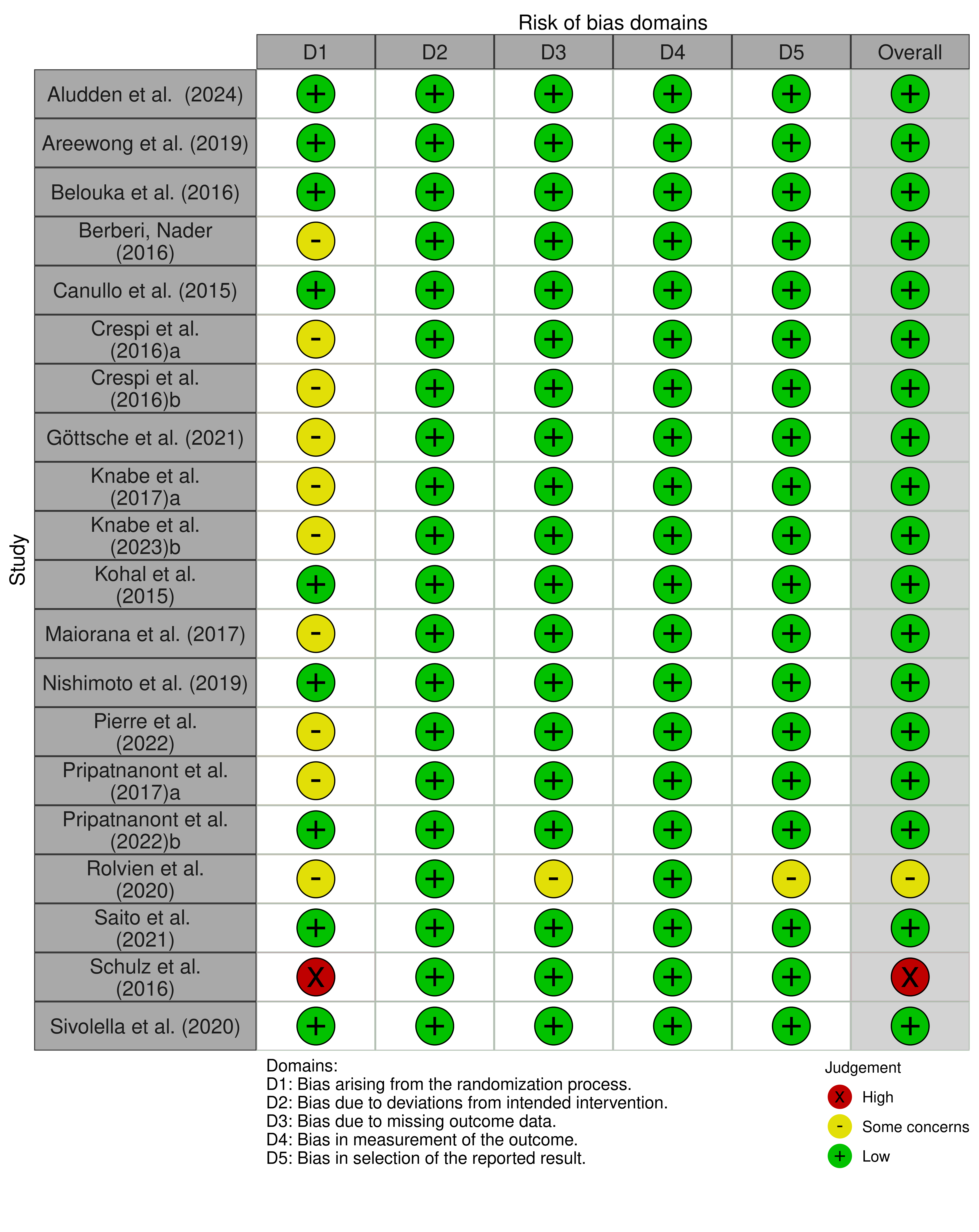

Supplement: Supplementary file 1 [file materials-18-00119-s001.zip › Supplementary file 2 - risk of bias assessment for human studies.png]
